# HelpResponder—System for the Security of First Responder Interventions

**DOI:** 10.3390/s21082614

**Published:** 2021-04-08

**Authors:** M. Cristina Rodriguez-Sanchez, Luis Fernández-Jiménez, Antonio R. Jiménez, Joaquin Vaquero, Susana Borromeo, Jose L. Lázaro-Galilea

**Affiliations:** 1 Electronics Department, Rey Juan Carlos University, 28933 Madrid, Spain; luis.fernandezj@urjc.es (L.F.-J.); joaquin.vaquero@urjc.es (J.V.); susana.borromeo@urjc.es (S.B.); 2 Center for Automation and Robotics (CAR) CSIC-UPM, E-28036 Madrid, Spain; antonio.jimenez@csic.es; 3 Electronics Department, Alcalá University, 28801 Madrid, Spain; josel.lazaro@uah.es

**Keywords:** real-time interventions, remote monitoring, indoor location, flame detection, video processing, Internet of Things, Location-Based Service

## Abstract

Firefighter’s interventions under dense smoke and flames are hazardous and ideally need an efficient in-advance geo-located actuation plan. The existing communication and sensing technologies should be customized, optimized, and integrated to better know the conditions (flame locations, air condition) before and during the rescue team’s interventions. In this paper, we propose a firefighter intervention architecture, which consists of several sensing devices (flame detectors, carbon dioxide air content) a navigation platform (an autonomous ground wheeled robot), and a communication/localization network (BLE IoT network) that can be used before and during an intervention in rescue or fire extinguishing missions even for indoor or confined spaces. The paper’s key novelty presents our integrated solution, giving some key implementation details and an intensive experimentation campaign in two real firefighter scenarios with real controlled fires. Results carried out in these real indoor scenarios are presented to demonstrate the feasibility of the system. A fire detection system is proposed to improve fire focus in real time and moving in confined spaces with no visibility and physical references. The results obtained in the experimentation show the proposal’s effectiveness in locating the fire focus’s position and orientation reducing time and risk exposure. This kind of location-aware fire integrated systems would significantly impact the speed and security of first responder interventions.

## 1. Introduction

Location and guidance services based on the current conditions and hazards play a critical role in emergencies. Accessing real-time information on what is happening and deciding how to evacuate people is vital, both for victims and for rescue personnel intervening in the evacuation/emergency. This information is essential in a fire emergency in indoor or large spaces (i.e., hospitals, universities, shopping centers, educational centers, galleries, and tunnels) where a priori information on the fire source and spread within the facilities is unknown.

In emergencies, flame detection systems quickly detect hazard situations that reduce the danger for human lives, limit the fire propagation, and minimize its negative economic impact. Fire transforms a closed space into a hostile environment [1,2,3]. Referring to specific numbers, 87 firefighters died in 2017 in the United States, 49 of whom were in emergency services [1]. Exposure to fire conditions, such as smoke inhalation, burns, excessive effort or stress, or being trapped, accounts for more than 60% of firefighter deaths, and caused more than 20% of fire injuries [2]. In particular, this investigation concerns localized fires in gallery-like geometries. This matter falls within various fire fields, such as fire safety in specific, confined places such as a parking lot, underground mining, or tunnels. The spread of fire and smoke circulation in a parking lot is an essential issue for people’s security, and the risk of death for people in such public areas has to be estimated. Numerical simulations treated this question in [4]. The general problem of fires in tunnels is also prominently featured; the safety matter is an old topic [5,6].

Thus, adding general preventive measures, such as fire detection and monitoring systems, would minimize the number of potential accidents. Unfortunately, current flame detection systems need to be close to the fire focus and are not always reliable because smoke and some features of some elements (colors or textures), such as fluorescent bulbs or lamps, do not necessarily indicate fire [7,8]. To propose a solution to this problem, all possible useful data during these situations must be collected and analyzed in real time.

There are some solutions to improve a firefighter’s services in emergencies [9]. In monitoring and sensing, students at the University of Missouri S&T have created FREAS [10], which sends data to the fire department during an intervention. It is therefore crucial to collect information from the environment in the moments prior to the intervention of fire fighters, and also prior to the autonomous navigation [11]. The information obtained from the environmental state is helpful for operational tactics. Moreover, to improve the environmental acquisition, the inclusion of images from the environment could offer relevant information. Most solutions usually use RGB cameras to explore the indoor environment [12]. However, those solutions cannot be applied indoors with no visibility.

This paper describes a platform to provide support services to Security, Prevention, Rescue, and Evacuation Services in indoor interventions. Our work’s main contribution is the detection and geo-location of the fire focus to establish the emergency team’s best access and intervention routes. We have oriented our research on locating the fire focus before the firefighters’ intervention, even when the fire is almost imperceptible, thus reducing exposure to risk and potential accidents, offering added communication and visibility levels where it is not available.

Firstly, We start with a review of current techniques. Secondly, the global description of our proposed architecture is explained: a Beacon Network, a Mobile Ground Autonomous Vehicle with an on-board fire detection infrared vision system, the protective firefighter equipment with embedded IMU sensors for position tracking, a real-time communication link, and a monitoring interface. Thirdly, we focus on implementing the safe and flame-aware intervention architecture explaining the fire detection system and the Mobile Ground Autonomous Vehicle (MGAV) for flame/fire focus detection. Fourthly, the tests and results in two scenarios to evaluate the proposal are explained: Alcorcon Unified Security Centre (USC) Fire Tower and Teresa Infrastructure (ILUNION, Brunete). It will discuss additional benefits derived from this new version’s usage at the end of this paper. Finally, the conclusion is presented.

## 2. State of the Art

The next subsections present a review of state-of-the-art monitoring, detection of fire focus, and location in indoor spaces related to interventions in emergencies and the absence of GNSS signal. Finally, our proposal is presented.

### 2.1. Environmental Monitoring

In monitoring and sensing using autonomous navigation collecting data devices, the researchers in [13] presented a mobile robotic olfaction system that allows the performing of online gas-sensing tasks in unknown open environments. This work’s drawback is that it does not apply air quality sensors (CO, CO_2_, NH_3_) to improve the intervention. The value of CO over 12.800 ppm is mortal within 1–3 min, and the HCN (hydrogen cyanide) at 180–270 ppm [1,2] were fatal within 1–3 min. Moreover, there are no cameras to obtain images of the environment and contrast the collected data. However, the model was attractive regarding the guiding in an unknown environment.

Another monitoring system for emergencies and disaster support called “Critical and Rescue Operations using Wearable Wireless sensors networks” is presented in [14]. This system showed the limitations of proposals based on adopting low power consumption wireless standards for body-to-body communications. This work used a dedicated routing protocol for disaster relief. The main drawback of these projects is that they were validated in simulated environments.

### 2.2. Image Processing for Flame/Fire Focus Detection

It is necessary to predict the origin of a flame before it spreads, creating a dangerous situation. Thanks to the increasing advances in neural networks applied to image processing, it is possible to train a deep neural network capable of detecting fire [15] in thermal images. However, it is necessary to improve this technique in those works to shorten the processing time and reduce false positives and false negatives.

Most solutions usually use an RGB camera. However, it is necessary to validate artificial vision algorithms in indoor spaces without visibility. For instance, a computer vision-based fire detection method is presented in [16], where a robust color model was developed to detect all candidate fire regions reliably. However, that work is based on RGB images and is not oriented or validated to operate in indoor spaces (without visibility because of the high level of smoke and humidity) in emergencies. Finally, it is not integrated with location and guiding systems for firefighters in emergency locations. In fact, we want to investigate solutions that can be adapted to the limitations and parameters associated with indoor. One of our research’s main objectives is to offer indoor solutions that offer added value to the limitations of outdoor solutions.

Another work-related with the use of thermal images is [17]. It obtains the characteristics to classify fire. However, this work is evaluated using a database with videos and images, not in a real scenario. The researchers in [18] proposed a new flame detection algorithm based on a saliency detection technique and the Uniform Local Binary Pattern (ULBP). Moreover, they used an exponential function with two parameters to model the flame area’s texture to reduce the number of false alarms. The proposed algorithm is based on images (photography) before applying the algorithm. Therefore, the experimental results are not applied in real time. Therefore, it is useful for off-line post-processing in situations where real time operation is not necessary. In [19], local binary patterns are used for solving flames detecting problems. Modifications were proposed to improve the flame detection quality using a Support Vector Machines (SVM) classifier with a kernel based on Gaussian Radial Basis Functions. It showed improvements in the time required to process the image and the detection parameters’ average accuracy. However, the processing time was too long to use it in real time.

### 2.3. Location Estimation for Navigation and Firefighter Tracking

On the one hand, regarding the navigation time in a burning building, researchers in [20] developed a platform to reduce the time with a multi-agent simultaneous localisation and mapping (SLAM) technique. The results indicated that the built map was more accurate than the map obtained using the conventional TSD-SLAM. Additionally, it builds the merged map more correctly by determining the proper parameters for online map merging. The problem is that SLAM is not the optimal solution when fire and smoke are present because typical SLAM revisits in closed trajectories cannot be done. It is necessary to explore other mixed solutions in this scenario [21].

On the other hand, there are some works [22,23] that describe rescue operations and security issues due to earthquakes, harsh climate, etc. Supplying location and tracking systems is essential to save firefighters’ lives during fire operations and speed up the rescue intervention. These works explained the deployment and monitoring of environmental parameters in outdoor environments. Future developments will include the implementation of the system in a heterogeneous scenario with obstacles. It will allow studying the behavior of the system reacting to different propagation of the IoT nodes signal and the obstacle avoidance movement for autonomous navigation systems and firefighters. Moreover, for beacon-based indoor locations, the most commonly used wireless technologies are WiFi, Bluetooth, and ultrasound [24,25,26], with ultrasound being the most accurate at centimeter-level RF-based solutions (WiFi and BLE), achieving accuracies of about 2 m. For instance, in [25], the authors used a network of beacons (fixed devices on the wall based on Bluetooth Low Energy) that remained connected to the device they wanted to locate, continuously estimating its position. However, this system’s location depends on many parameters, such as interference, temperature, obstacles, and the signal’s range. In the case of using the Zigbee positioning system in real Non-Line-of-Sight (NLOS) conditions, such as in large buildings with multiple walls that the RF signal has to penetrate, the accuracies dropped significantly to a minimum positioning error of 2 m [26]. There are other approaches to determine locations in indoor environments, such as those using LoRa (Long Range). Despite its robustness in open areas to provide stable and efficient communication, the use of Lora technologies in indoor environments is in an initial state and requires further study regarding the effect of signal propagation obstacles. Inertial sensing devices have many advantages, such as making sense independent from the environment. Many positioning proposals include the INS (Inertial Navigation System) approach, known as PDR (pedestrian dead-reckoning) when applied to monitor people while walking.

An INS/PDR location solution uses inertial sensors, such as accelerometers and gyroscopes, usually integrated into an Inertial Measurement Unit (IMU). Data obtained from these sensors need post-processing, which includes integration calculations, gravity compensation, bias removal, and filtering to get proper motion (3D position) and pose (3D orientation) information [27]. There are numerous projects in this area. New Round-Trip Time-of-Flight technology such as Ultra-Wide-band (UWB) ranging is also a good complement for improving positioning accuracy at a decimeter error level [28]. Additionally, to remove the drift in inertial estimations, such as the principal building direction approach (iHDE and MiHDE) and the simulation of IMU signals for particular IMU fixes on a person’s body [29]. Other heuristics using an IMU and beacons (RFID, WiFi, BLE) are combined in [30,31], which decreases the positioning error to less than 1 m. They compare different algorithms for step detection, stride calculation, and positioning, achieving maximum errors of 5% of the total travelled distance and using a low-priced Inertial Measurement Unit (IMU). Finally, several improvements are proposed in bias estimation from IMU sensors using the refined version of ZUPT (Zero velocity update at foot stances) and MARU (Magnetically aided calibration) [32].

In conclusion, the devices used by emergency personnel in indoor locations lag well behind the already existent technological and scientific progress regarding navigation devices, image processing for fire focus detection, decision-making, and significant data process. The proposed monitoring system described in this paper will facilitate autonomous monitoring and decision making in real time to minimize risk situations and possible accidents derived from the lack of awareness of the emergency locations, the state of the environment, and the monitoring of the paths taken by firefighters. These provide valuable information in an emergency intervention and impact firefighter response times in safety-critical missions.

## 3. Global Description of the Architecture

### 3.1. Our Contribution

The objective is to manage relevant information from the environment to decide an intervention concerning the firefighter’s tactics and use graphic interfaces for emergency ACP (Advanced Command Post)—the administrator of interventions and navigation tactics. Our system can provide valuable information in an emergency intervention, and its use would impact the firefighters’ response times, operational timeframes for firefighters, and avoidance of potential accidents.

This platform has been evaluated in two confined (indoor) spaces and an open space of approximately 70–130 square meters, simulating a small tunnel or small gallery, which reduces the response time in 3.02 s less. It has also been tested in conditions of absence of a GNSS (Global Navigation Satellite System) signal, with smoke (without visibility), and with high levels of temperature and humidity: a solution for cold fire (simulated fire) and fire (real fire). We chose those scenarios because there is no visibility in an open space (single space) in the presence of smoke. If we add to this the fact that there is no reference element (walls, stairs, columns, etc.) in the environment, the disorientation of firefighters or victims will be even greater. The aforementioned physical elements serve to provide a specific reference in the absence of visibility. When there is none, it is necessary to offer solutions to help orientation and navigation.

For this purpose, where there is no visibility, an autonomous navigation vehicle called a Mobile Ground Autonomous Vehicle (MGAV) has been developed, incorporating thermal image acquisition and using artificial vision algorithms to detect possible fire focuses in an emergency. The inclusion of thermal cameras and the automatic analysis of its images with thermal information are oriented to improve monitoring with images and support the emergency team in such high-risk situations. Moreover, MGAV can navigate among avoiding obstacles using ultrasonic sensors. This solution is based on an IoT sensor network to provide communications to the outside and collect and send environmental information to the ACP. Our biggest challenge is to provide good results with images taken in motion, in videos, and real processing. Most solutions offer tests with a static camera at a fixed point and with photos. However, in this work, we have deployed and evaluated the system using a moving camera.

Communications and acquisition of environmental parameters for the MGAV have been implemented through this IoT network using a wireless Bluetooth Low Energy (BLE) protocol. According to the requirements of the emergency services, the next main parameters have been chosen to help operational tactics: environmental temperature, toxicity in the air, and CO concentration. The sensor network components (beacons) also include temperature and CO (Carbon dioxide) concentration measurements and flame detection. This sensor network must be deployed at the facility as part of its security infrastructure. We hypothesize that the beacons network is deployed in the environment and that there is an a priori known map of the space. The firefighters usually operate with maps of the areas to be intervened to take decisions. We will assume that the MGAV will have to reach at least as far as the point where the fire source is detected. With the information of its position through the sensor network and the camera’s orientation, it will be possible to know the location. This information and the map known a priori complemented with the IMU will optimize the firefighters’ navigation.

Our objective in this work is to investigate a platform that responds to minimize risks in an intervention by validating our architecture and algorithms improving the information about the scenario reducing time of exposure to minimize risks. Therefore, previously and during the intervention of firefighters, it would be capable of monitoring and operating indoors with hostile conditions associated with an emergency [13]. In the design and development of the proposed system, the recommendations and needs of the APTB (Professional Association of Fire Fighters Technicians) have been followed. More precisely, concerning the safety and accessibility of buildings in emergencies and the viability of monitoring, the guidelines developed in the Basic Documents of the Spanish DB-SUA Technical Code have been taken into account, following the RD393/2007 regulation [11].

### 3.2. The Monitoring System

**Figure 1:**
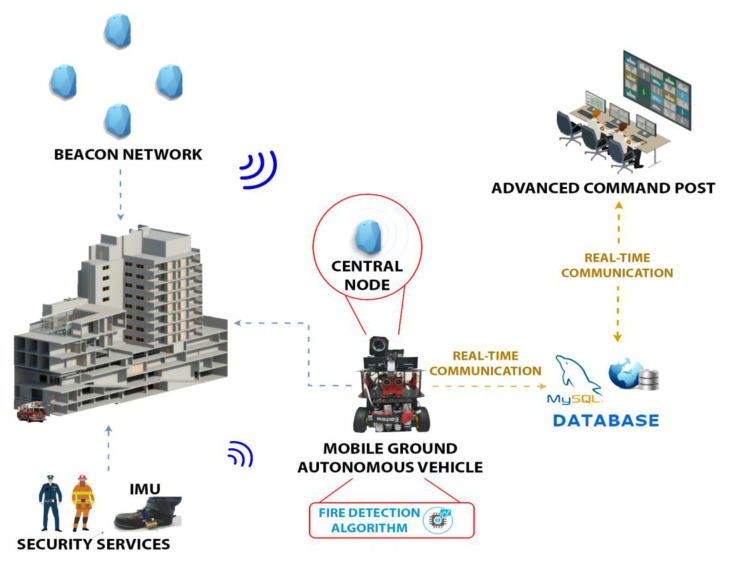
Diagram of the proposed monitoring system for indoor first-responder interventions.

The approach proposed in this paper consists of a system capable of analyzing the conditions in which a hostile environment is found before performing the intervention by the first-responder team. These could be very useful for giving real-time feedback during the intervention process. Figure 1 shows the complete architecture consisting of the following modules: (1) a Beacon Network for ranging/sensing, which is deployed in smart buildings, (2) the Mobile Ground Autonomous Vehicle (MGAV), geo-located by ranging with the central node and including (3) an onboard fire detection infrared vision system, (4) the protective firefighter equipment with embedded IMU sensors for position tracking, (5) a real-time communication link, and (6) an Advanced Command Post Interface (ACPI).

The Beacon Network (1) distributed over an interior space will be deployed in the indoor environment (Nordic Semiconductor nRF52840 PDK [33]). They are configured in a star topology, in which a device or central node collects data from the peripheral beacons or nodes. These peripheral beacons could collect environmental parameters such as temperature or CO concentration and detect flames’ presence in their surroundings. This proposal will use this sensor network used to provide communication to send information and location. This network will be used by the MGAV, which includes the communication module to navigate in the indoor environment. The development of beacons is integrated on an ATEX box to include and protect the electronics from operating in the evaluated scenarios’ conditions.

The main contribution of the MGAV is the integration of a Fire Detection System to locate the fire focus emergency with real-time video capture and processing equipment indoors and without visibility by the fire smoke. The MGAV is based on [34] and uses sensor networks and three ultrasonic sensors for free space detection to navigate the environment. The ACPI interface is used to monitor the critical information collected from the environment showing the path followed by the MGAV to find the point close to fire focus emergency using a typical dashboard. We hypothesize that the beacon network is deployed in the environment and that there is an a priori known map of space. Our research is oriented to facilitate the system to detect points of interest and provide the most optimal route tracking to the emergency services. This type of service usually operates with maps of the areas to be intervened. Our objective in this work is to investigate a platform that responds to minimize risks in an intervention by validating our architecture and algorithms. In the future, our objective is the ability to deploy this platform in an infrastructure that is not known. The next section will explain this paper’s main contribution: the Fire Detection System using the data collected by the MGAV.

## 4. Implementation of the Safe and Flame-Aware Intervention Architecture

We integrated into the platform the next modules to validate the fire detection algorithm and send information about the emergency’s environmental state.

### 4.1. Fire Detection System

**Figure 2:**
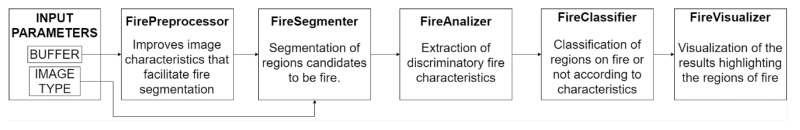
General block diagram of the fire detection system.

**Figure 3:**
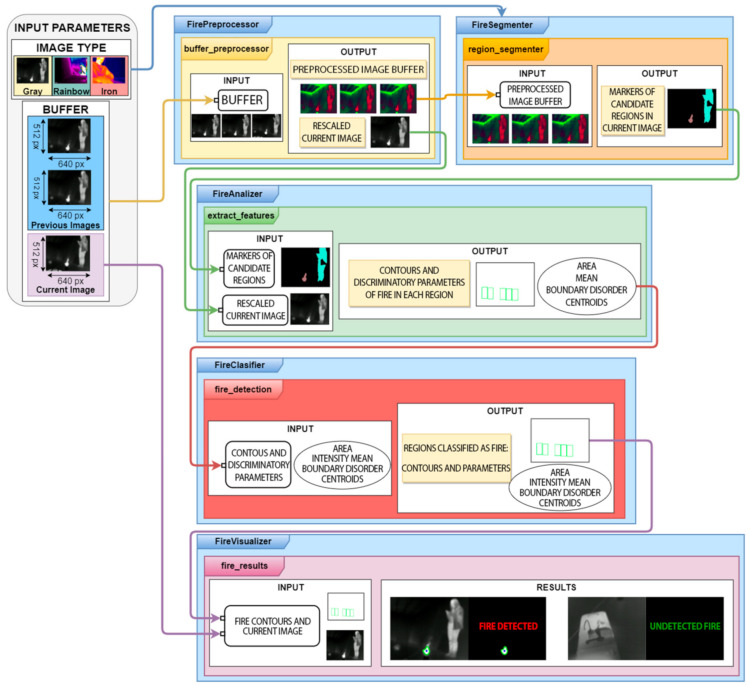
Fire detection algorithm schematic.

In this section, we will explain the fire detection algorithm for the MGAV. The FLIR A65 [35] camera was chosen as a thermal imaging temperature sensor for fire detection and condition monitoring. This camera offers comprehensive visual temperature monitoring. A UDOO XII ADVANCED PLUS [36] module has been used to process the video captured by the FLIR A65 camera. The computer vision algorithm designed for fire detection in thermal imaging can operate with video sequences in low visibility conditions. Figure 2 shows the scheme that describes the implemented algorithm operation’s operation; Figure 3 explains it in more detail.

The fire detection algorithm performs temporal analysis and color filtering to detect fire candidate regions in the image. The camera has a robust design that can withstand harsh conditions, and it can operate in temperatures up to 140 °F (60 °C). The temperature range measured on objects with this camera is −40 °C to 550 °C (−40 to 1022 °F). We extract features to discriminate fire from other similar regions such as area, boundary disorder, and average intensity measured for each obtained region. The algorithm receives as input the type of image to be processed.

The first detection stage (FirePreprocessor), see Figure 3, improves the image characteristics to simplify the detection tasks in the following stages. Firstly, ROIs (Regions Of Interest) are established along with rescaling with linear interpolation. The objective is to decrease the number of pixels to be processed. Secondly, Gaussian smoothing is the traditional denoising method. This method allows the transformation of the images to the HSV (Hue Saturation Value) color space, removing the noise. Finally, the image contrast has been enhanced by combining two techniques: CLAHE (Contrast Limited Adaptive Histogram Equalization) and ImageEnhace. Contrast with a factor of 1.5.

Once we have the processed images, the segmentation of candidate regions is started (FireSegmentation). The first method is a color filter based on the HSV format to get the hot elements located at the scene. The values are as follows:

*Colour_min = [0,0,105]* *Colour_max = [10,50,255]* (1)

Those parameters were obtained from several tests with the camera in an indoor space with smoke. We have deployed and evaluated the system using a moving camera in this work. Therefore, image processing is more complicated than when using static cameras.

At this point, the elements of the buffer that have got this previous filter have been obtained. Temporal analysis is performed. This analysis will identify the objects that are presented in all the images. Moreover, it has been taken into account that the fire is a dynamic element. The hypothesis is that the next elements of the scene are static—the volume and movements do not change. Once the binary images are obtained from the color filter, Equations (2)–(4) describe the subtraction of images:

*diff_2_1 = cv2.absdiff(image_binary_current, image_binary_previous1)* (2)

*diff_2_0 = cv2.absdiff(image_binary_current, image_binary_previous0)* (3)

*diff_image = cv2.absdiff(diff_2_1, diff_2_0)* (4)

**Figure 4:**
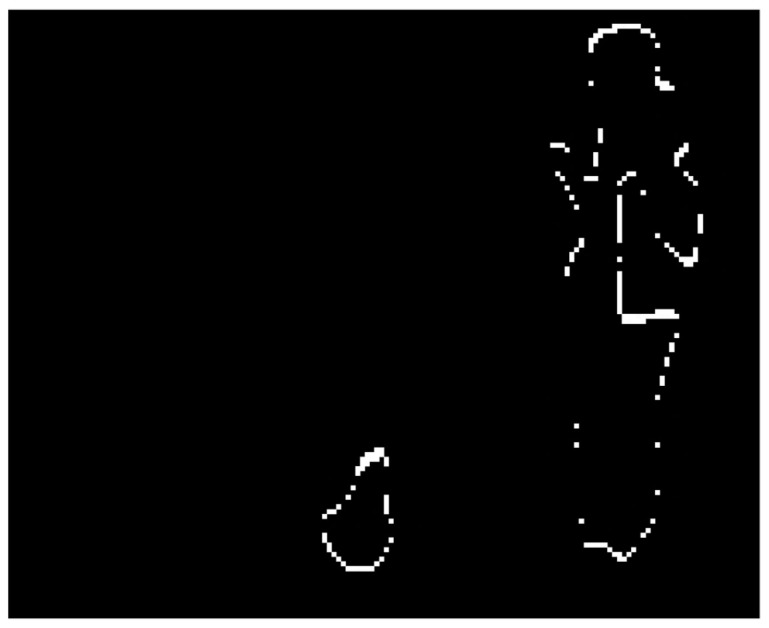
The pixel movement of the image.

The result shown in Figure 4 is a binary image. The pixels of the objects that appear in the three images are the foreground pixels. They also have varied positions.

**Figure 5:**
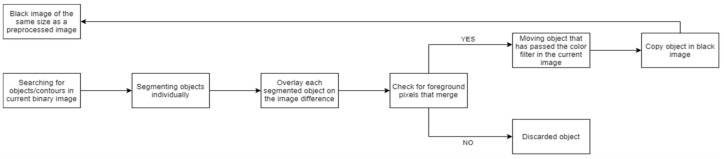
Block diagram of the pseudocode for checking the regions of the current image to which moving pixels belong.

In this stage, the next step is getting and checks the regions of the current image. The target is to detect flame or fire where the pixel in movement belongs, see step at Figure 3 and the result in Figure 4. These steps are explained in Algorithm 1 and the diagram below (Figure 5). This process detects elements in a movement that have passed the color filter. These objects will be used as seeds. The seeds are a related group of foreground pixels to make the Watershed. Then, the candidate regions are obtained to correspond to fire zones.
**Algorithm 1. Pseudocode for for checking the regions of the current image to which moving pixels belong.**1. seed_objects_image = np.zeros(diff_image.shape, np.uint8) 2. contours, = cv2.findContours(bin_acutal_image,cv2.RETR_EXTERNAL,cv2.CHAIN_APPROX_SIMPLE) 3. # Each connected region of the original image is overlapped to the image difference4. for cnt in contours: 5. # The image with the connected region is created. 6. region_image = np.zeros(diff_image.shape, np.uint8) 7. cv2.drawContours(region_image, [cnt], −1, 255, −1) 8. # Image with foreground pixels being those that both in the image and in the region image are foreground 9. movement_region_image = cv2.bitwise_and(diff_image, diff_image, mask = region_image) 10. # if there is foreground pixels → Si hay pixeles de primer plano → Add region to image 11. if not np.all(movement_region_image = 0): 

The third stage focuses on obtaining the regions that are the real fire or flame; it is necessary to obtain their characteristics (FireAnalizer) and to discriminate small regions that are not of interest (the average intensity). The objective is to rule out regions that have passed the color filter but are not strong enough to be fire. The boundaries disorder is a parameter to analyze objects discarding objects with a regular shape of [37], centroids, and contours.

A four-stage method (FireClassifier) is developed to consider the following discrimination tasks in the image processing:

Discrimination by the region’s shape: for this kind of classification, the area and boundary disorder parameters are used. The regions must exceed established threshold to be considered a fire or flame.

Discrimination by intensity: as described above, to obtain the fire regions, we applied a color filter.

The next ranges are used to determine the Boundary disorder, Area, and Intensity mean:

*0.7 < Boundary disorder < 0.95 300 < Area 130 < Intensity mean* (5)

The fire detection depends on the conditions, but we have used some parameters (thresholds) to validate two different scenarios without changing these parameters in the paper. It relates to the list of parameters that should be adjusted in different environments. In this study, there are confined (indoor) spaces and an open space of approximately 70–130 square meters, simulating a small tunnel or a small gallery: a 120 m^2^ gallery with an open space, for example used for parking. We previously analyzed different thresholds oriented to those scenarios. In this case, we use the following parameters: boundary disorder, intensity average, and area size. Moreover, we based the experiment on the hypothesis that in those kinds of scenarios, the context would not change significantly, so the algorithms’ results would be similar.

Regions meeting these conditions shall be considered as flames and shall be marked in the current input buffer image. In the last stage (FireVisualizer), the algorithm stores the characteristics of the regions classified as fire with their contours. These regions and contours are transferred to their representation.

The metric used is important. Some works [38] in classification performance metrics were based on the binary confusion matrix. Below, the methodology to evaluate the results is explained. Firstly, we evaluate the accuracy of the process, detection metrics, and processing times. These were used as an evaluation metric. In a pattern recognition task, we needed to define the true positives and the false positives. We measured the accuracy of the flame detection at the region level and used the IoU (intersection over union) for our evaluation metrics. Their union divides the intersection of the detection result and ground truth, which is the detection accuracy. The following equation was used to calculate the IoU:

(6) $$IoU=\frac{detection\;results\;\cap^{}\;gound\;truth}{detection\;results\;\cup^{}\;gound\;truth}$$

**Figure 6:**
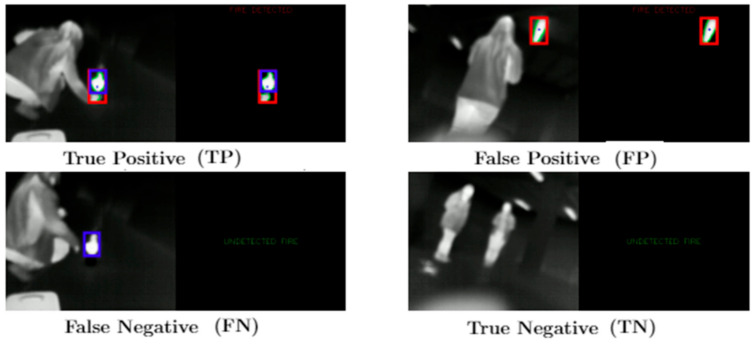
Definitions of TP, FP, FN, and TN.

If IoU > 0.5 between the predicted box and the ground truth box, the predicted box will be a “true positive (TP)”; otherwise, it will be a “false positive (FP).” The “false negative (FN)” and the objects that the model has missed out (Figure 6) were measured. The predicted bounding boxes are red, and the ground truth boxes are blue.

The metrics we have used to measure the detection efficiency of the algorithm are defined in the following equation:

(7) $$Accuracy=\frac{TP+TN}{TP+FP+TN+FN}$$

The metric accuracy does not work well when classes are unbalanced, as it is in this case. For problems with unbalanced classes, it is much better to use precision, recall, and F1. These metrics give a better idea of the quality of the model:

(8) $$Precision=\frac{TP}{TP+FP}$$

(9) $$Recall=\frac{TP}{TP+FN}$$

(10) $$F1\;Score=\frac{2\cdot Precision\cdot Recall}{Precision+Recall}$$

### 4.2. Mobile Ground Autonomous Vehicle (MGAV) for Flame/Fire Focus Detection

**Figure 7:**
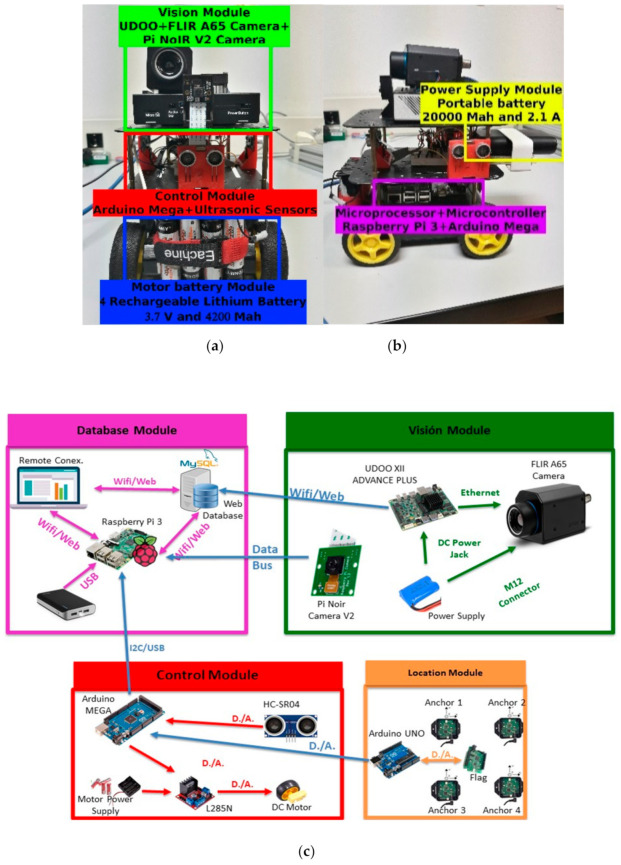
Autonomous indoor navigation device. (**a**) Frontal, (**b**) lateral, (**c**) connection diagram.

The data acquisition system also includes real-time video capture, environmental monitoring, and processing equipment coupled to the mobile ground autonomous vehicle (MGAV). The MGAV is based on [33] and uses three ultrasonic sensors to recognize the environment, with sonar operation controlled by an ATmega328P microcontroller. We include more sensors to improve navigation and acquisition. Figure 7a,b shows the components and deployment, and the connections between them are shown in Figure 7c. The MGAV can navigate within the confined enclosure without user interaction. It can monitor the environmental parameters to detect the focus of the emergency based on network deployment.

The description of the modules is as follows:

Vision module: as we described in a previous section, the UDOO contains the intelligence to detect fire by using artificial vision.

Database module: it allows access to monitored data related to images, environmental data, and data of interest at an intervention. We implemented an external database server for remote data management based on a MySQL Server 5.7.29 installed on an Ubuntu server 18.04 LTS (Long Time Service) machine. The access to the database content uses HTTPS and secure authentication. Therefore, only authorized users can obtain access. All stored data is time stamped, and it is associated with a specific experiment, allowing a comparison of the result between the different tests.

Control module: the MGAV can navigate among avoiding obstacles using ultrasonic sensors included at this module. The MGAV has been equipped with HC-SR04 ultrasonic sensors; an ATMEL processor; the TT Micro DC Geared motor—capable of rotating at 160 rpm without unload at 6V voltage; a motor driver based on the L298N integrated circuit, a dual full-bridge driver; and finally, protection diodes and an LM7805 regulator which supplies a 5V supply voltage to the L298N IC and also to external sensors.

The location of the beacons will be developed for each scenario to locate firefighters. This means that the robot has distance sensors (ultrasound) to overcome inaccuracies in the absolute location. An RF52840 PDK was used [33]. Each node can be integrate a different environmental sensor. For this research, we have validated the onboard unit using a KY-026 (flame sensor), the SoC nRF52840 PDK temperature, the LM35 (environmental temperature), and the MQ-7 (toxicity in the air, CO concentration sensor) [39]. We have defined the next equations to calculate the values of measured risk. The AnalogReadT [see Equation (6)] is the value registered from the SoC nRF52840 PDK temperature. The AnalogReadE (see Equation (7)) is the value registered from the LM35. The minimum conditions establish an exposure time of fewer than 30 min for the following parameters: air temperature of 60 °C, carbon monoxide contents of 350 ppm. A CO value over 3000 ppm and an HCN (hydrogen cyanide) value over 300 ppm are fatal. These values will be useful to evaluate the critical factors in the air during intervention according to the normative described in [1,2,3]:

(11) $$\mathrm{Temperature}\;\left[{}^{\circ}C\right]=\mathrm{AnalogReadT}\cdot \frac{5000\;\mathrm{mV}}{4096}\;\cdot \frac{1\;{}^{\circ}C}{10\;\mathrm{mV}}$$

(12) Rs[Ω]=RL·5−VinVin=1000Ω·5V−(AnalogReadE·54096)AnalogRead·54096

(13) $$\;CO\;concentration\;\left[\mathrm{ppm}\right]=233.9\cdot {\left(\frac{Rs}{5463}\right)}^{-1.4}$$

## 5. Results

For this project’s development, we used two scenarios to evaluate the proposal: Alcorcon Unified Security Centre (USC) Fire Tower and Teresa Infrastructure (ILUNION, Brunete). Those places are specifically designed for the training of security services personnel in emergencies. The main concepts that we highlight are that the fire is found, the firefighters can be guided to it after it is found, and that this procedure will save valuable time. Each evaluation was carried out over 3 days (4 h per day) thanks to the collaboration of those entities.

### 5.1. Test Site 1: Alcorcon USC Fire Tower

**Figure 8:**
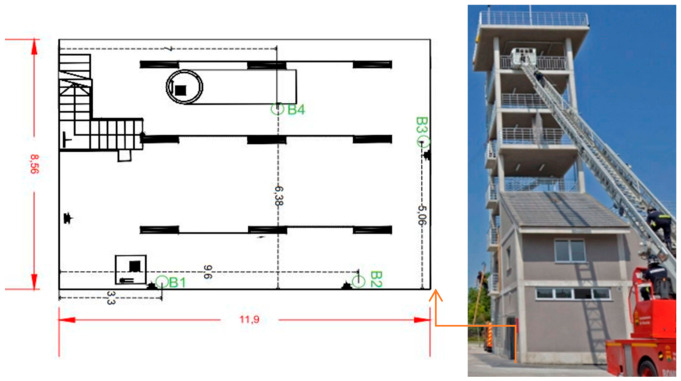
The confined space for the evaluation with the support of the USC-Alcorcón.

First of all, we describe the space of the evaluation. In Figure 8, there is a map with the confined spaces’ dimension for the evaluation and the beacon’s network location (B1→B4). It is the basement of the USC. Secondly, to carry out this first test, a cold smoke-generating machine is required to reduce visibility and worsen environmental conditions to test the communication between the beacons. Table 1 shows the RSSI measurement results among peripheral nodes and the central node. The power configured in the network for each of the distances is indicated.

**Figure 9:**
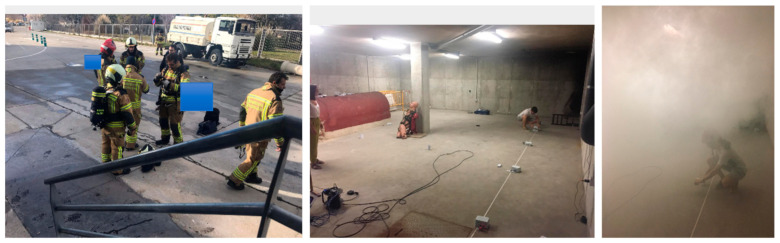
Pictures were made during the evaluation with the firefighter teams of the USC-Alcorcón (Unified Service Center).

The description of the evaluation is next. After the intervention of the MGAV, the firefighter team could start the interventions (see Figure 9). The firefighters moved along the enclosure perimeter according to emergency intervention procedures and the focus fire detection results. The location of the beacons and the points are deployed, taking into account the coordinates that could guide firefighters in a future development using the interface for the ACP. Additionally, our system offered environmental data, a map, and a recommendation path to navigate the enclosure perimeter in previous firefighters’ intervention. With this evaluation, the ACP could optimize a firefighter route with information about the fire focus’s location and environmental conditions (moderate temperature and toxicity in the air). This route showed the point of fire focus and the points to explore the environment to find victims.

In all cases, RSSI is worse in cold smoke, between 1 and 5 dB on average. It is interesting to program the beacons at their lower transmission power when used in emergencies with adverse environmental parameters [40], such as smoke, to minimize signal strength losses. The theoretical values of RSSI are calculated from [41]. Taking n = 2 for free space [42] and varying A, the error between the theoretical and the experimental calculation is close to 1%.

The data can be extrapolated from these measurements, adjusting regression curves to obtain the maximum distance between nodes according to the minimum RSSI supported by the beacons, which is −96 dBm. Firstly, comparing the RSSI signal for the same power with and without smoke, one can expect that the smoke case would make the signal worse, but this is not always the case. The peripheral node antenna faces better, with the antenna of the central node improving the RSSI signal. Each one is at a different distance from the central node, so they have different RSSI values. They are all configured to work below −92 dBm in absolute value. When they reach 92 dB in absolute value, they switch off because they cannot operate with such low RSSI quality. That is why we have set the transmit power to the maximum allowed by the manufacturer: 8 dB.

**Figure 10:**
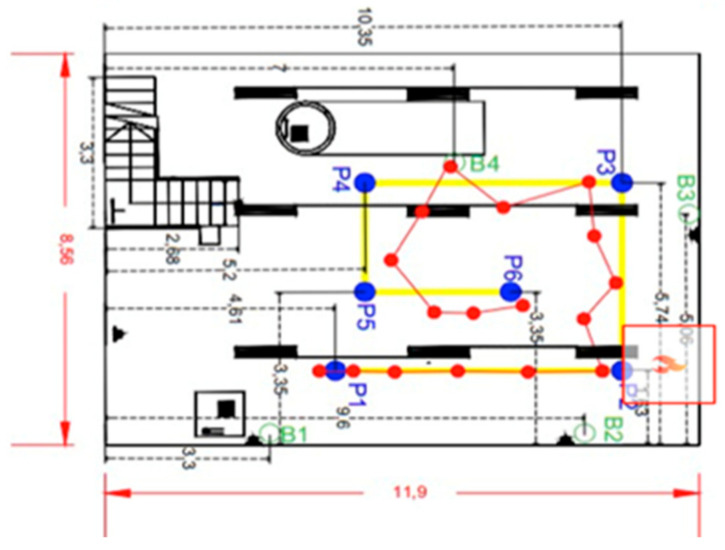
USC map with location results.

Secondly, it will explain the process to obtain the fire’s location and orientation once detected. In Figure 10, the used point of the space to deploy the beacons has been represented.

Hence, this determines the positions in which the beacons were deployed (B1, B2, B3, and B4). The purple dots and yellow lines show the ideal path to follow to navigate the space. It showed the followed path (red line) of the MGAV to explore the indoor environment and locate the fire’s focus. Several points were chosen to emulate the movement of the MGAV (P1, P2, P3, P4, P5, and P6)—see Table 2 according to the received information during the intervention. The actual path is the red line that joins the points P1 to P6.

The firefighter has a BLE device in their pocket and an IMU board in their boats. The error location based on the previous information and the Beacon Sensor Network location obtained in these different tests ranges from 40 cm to 70 cm. The experiment carried out is in the area described previously, which has a high density of nodes. Indeed, the precise location results (less than 1 m) cannot be obtained only with BLE, but in our case, it is integrated with IMU, which has high precision in the short term, so by merging BLE + IMU/PDR, it is possible to reach 0.5 m, even in large buildings. The experiments have obtained excellent accuracy, with errors of about 0.5 m, enough to guide the robot and the firefighters. This means that the firefighter can pass through doorways, and the robot has distance sensors (ultrasound) to overcome inaccuracies in the absolute location. Those results are used to locate in the map the estimation of the fire focus (close to P2).

We performed two tests to ensure the firefighter would get to the fire source based on the orientation of focus of the emergency based on previous results. One was made without our system, and one with our proposal. In the test, the firefighters arrived 3.02 s earlier at the fire source thanks to the proposed system than without it. This test concludes the camera’s correct operation and the algorithm in a real environment where there are elements with high temperatures.

**Figure 11:**
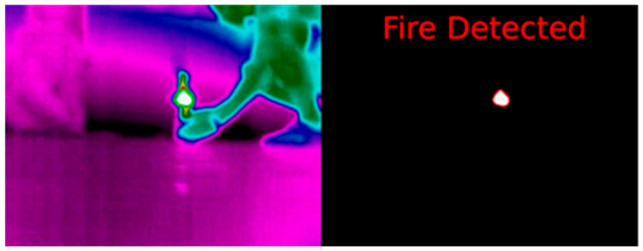
Result of the artificial vision algorithm for fire detection at USC.

The next step was introducing the MGAV inside the space using the previous communication based on beacon networks. The MGAV was implemented following the Section 4 design and the Location Block and planning for the MGAV code. The MGAV follows the beacons in the next order B1→B2→B3→B4. It was guided and sent the collected environmental data and images to validate fire detection by artificial vision. To emulate the fire, we have used a gas stove capable of producing a high concentrated temperature, which was used to generate the focus of the fire.Following the methodology described in Section 4.1, Figure 11 shows the algorithm’s results detecting the flame without failure. Table 3 presented the results of the characteristics, detection metrics, and processing time. The FLIR A65 produces stunning 327,680 pixels (8-bit 640 columns and 512 rows images streamed) thermal images with low noise and can show temperature differences as small as 50 mK. These allow to easily tracking temperature changes, stream thermal images at up to 30 Hz directly to the system, and instant data analysis in UDOO board. At distances of less than 7 m, the temperature that the gas stove generated was high enough to exceed the level established in the flame detection algorithm.

It should be noted that the camera can correctly differentiate the focus from the rest of the scene. Moreover, an element is used that generates fire in a limited region, which does not simulate a real fire, but the detection algorithm is appropriate for the test. With this test, we successfully detected small simulated fires; the next section shows the results of a test with a larger fire.

### 5.2. Test site 2: Teresa Infrastructure

**Figure 12:**
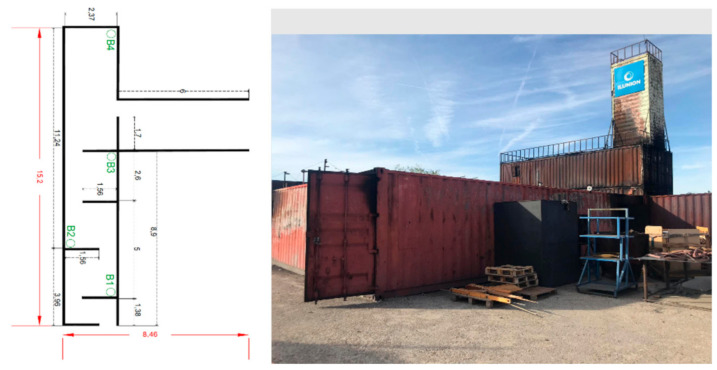
Representation of the MAP for the Teresa infrastructure evaluation and pictures of the scenario to prepare an intervention with real fire supervised by the Teresa group; the location is in Brunete (Madrid, Spain). On the left is a map showing the dimensions of the interior space, which also shows the deployment of the beacons. On the right, there is a picture of the outside.

Figure 12 shows a map with the dimension of the confined space in the USC and the beacons’ location design. The gallery has three exits and two entrances, so it is important to define which one is closest to the fire.

**Figure 13:**
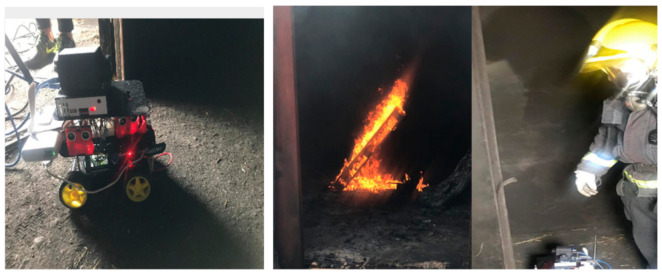
Pictures made during the evaluation in the CUS with the Teresa firefighter teams.

In this case, the test is carried out in a small tunnel-like space with metal walls. In this test, a real fire is lit in one of the tunnel rooms (see Figure 12 and Figure 13). This test allows the system to be validated in a new environment with different test parameters, for instance, higher temperatures (up to 110 °C) and hot and real smoke. The transmission power of 8 dB has been used, which is the most appropriate, as mentioned before. Table 4 shows the RSSI measurement results among peripheral nodes and the central node. The power configured in the network for each of the distances is indicated.

The row “Average” indicates how the hot smoke affects the RSSI signal, reducing it by 2 dB less. The hot smoke also affects the mode and the median except for the mode of beacon 2. The standard deviation and the variance improve their values in all beacons in the presence of smoke. Those results are expected because the hot smoke worsens the signal quality and emphasizes the importance of using the maximum transmission power not to lose the connection between nodes. This is related to the fire detection since at a 50 m distance from the fire’s focus, there was no vision and high humidity and smoke levels, with the artificial vision acquisition system in this scenario offering satisfactory results. The results in [43] verify the behavior of the algorithm shown in Table 5.

The evaluation of the results has been used the same methodology for the detection metrics and processing time described in Section 4.1. Table 5 shows the video’s characteristics made in Teresa applying the algorithm to the received video from MGAV. Table 6 shows the video’s characteristics made in Teresa applying the algorithm to the received video from MGAV.

**Figure 14:**
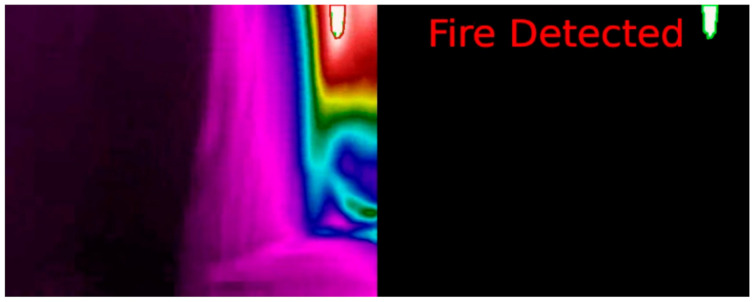
Erroneous detection of fire in the Teresa test.

**Figure 15:**
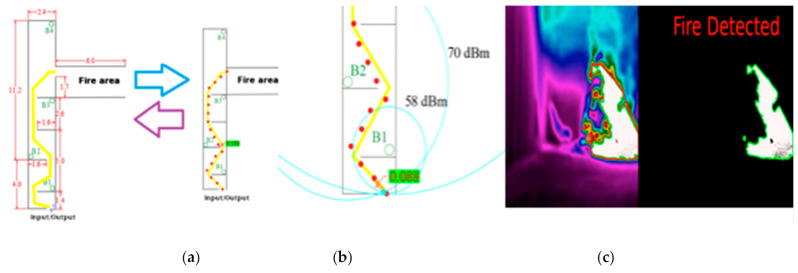
Map of Teresa with the real trajectory of the firefighter and the points detected by the IMU. (**a**) Result of the trajectory of the firefighter IMU+RSSI. (**b**) Correction of a point by the RSSI algorithm. (**c**) Result of the vision algorithm for fire detection obtained in the Teresa test

It has been confirmed that there are only false positives in those videos where there is fire; accordingly, there are temperatures above the threshold of 90 °C, and the fire is not the focus of the scene. In this case, the temperature and color of that area causes a failure in the algorithm. In these cases, the algorithm detects fire in areas on the image that are not on fire but have a high temperature. In these situations, there are small elements in the scene that the algorithm detects as fire. The algorithm should ignore these elements because they may be lights, reflections, or other elements, which are not fired and should be eliminated as a point of detection. An example of them is shown in Figure 14. This method’s great advantage consists of the low number of false positives, even with a camera onboard an MGAV, together with the reduced processing time. This value could be improved to ensure reliable results. It is noted that the proposed algorithm works best when the movement is in a straight line; when the error is at a maximum, the movement is in curves with pronounced changes of direction. Figure 15 shows those results where we could extract the location of the focus base: (a) is the point where the robot was located in the section, and (b) is the detection point.

Related to the navigation, in this evaluation, the MGAV followed the beacons in the order B1→B2→B3→B4. Figure 15a shows the system’s position at each point and the maximum error respecting the actual trajectory, which is 0.330 m (see Table 7). Moreover, thanks to the location’s support using the Beacon Sensor Network technology, it is calculated from a distance between peripherals beacons and the robot beacon, using a trilateration algorithm. Again, the experiment carried out in this subsection is conducted in a small area with a high density of nodes. Indeed, the precise location results (less than 1 m) cannot be obtained only with BLE, but in our case, it is integrated with IMU, which has high precision in the short term. Thus, by merging BLE + IMU/PDR, it is possible to reach an accuracy of 0.5 m, even in large buildings. Although the experiments have obtained excellent accuracy, with errors of about 0.5 m, it is enough to guide the robot and the firefighters. The focus was close to B3.

The next evaluation was to monitor the firefighter steps following the beacons B1→B2→B3. The firefighter has a BLE device in their pocket and an IMU board in their boats. This monitoring done using the selected IMU location algorithm [30] provided a real-time position of the firefighter for the ACP. The reference axes for the table are those shown in Figure 15 (a—Real trajectory of a firefighter) (“x” positive to the left and “y” positive upwards). The error described is the perpendicular measurement of the firefighter’s trajectory, with the point representing the step’s location (see Figure 15a). The RSSI-based location algorithm uses a spherical positioning system [42] that simulates the radio circumferences of a distance calculated according to the RSSI from each beacon. When two or more circumferences intersect, it provides the position of that point, as seen in Figure 15b. The error correction is made only in the indicated point. It is observed that the error of the new point provided by the algorithm is deficient, namely 0.088 m. In an indoor environment, there are situations of NLOS that decrease the signal strength by at least 20 dBm, and therefore, introducing many errors in positioning, as the circumferences do not intersect at the same point [44]. As can be seen in the results obtained, we could improve the positioning achieved by the IMU location device by applying the RSSI correction algorithm, being able to correct an error of 0.330 m at values improved 3–4 times.

To conclude, we performed two tests for two firefighters (four in total) to ensure that the firefighter would get to the source of the fire. One test was done without our system, and the other one with our proposal. In those tests, the firefighters arrived 6 min (medium) earlier at the fire source thanks to the proposed system (see Figure 13) as compared to without it, taking into account the dimension and map of Figure 12.

## 6. Discussion

This section will discuss the main issues and differences with other solutions in the context of the research paper presented according to the results explained above. This work’s objective was to provide frontline firefighters with an optimal fire rescue location strategy based on fire scene information (including ignition points, environmental parameters, and locations of firefighters). This information is obtained using network sensors and autonomous agents. Therefore, the firefighters can concentrate their efforts on the fire focus to quickly bring out the rest. This study is believed to innovate in location and monitoring firefighter interventions to improve methods in indoor operations.

Table 8 shows a sum-up comparison of the main contributions in the field of the research described in this paper. In the works [9,10,11,12,13,14], there is a lack of solution applied to indoor environments without visual information because of the smoke of fire and absence of GNSS signals. Our proposal’s system predicts the origin of the flame, the fire focus, thanks to the models that have been integrated into the algorithm explained in Section 3. In contrast with [16], the experimental results indicate that the developed processing method on thermal images in false color RAINBOW is a suitable solution for the fast detection of fires with a high accuracy rate in indoor spaces with smoke and no vision.

The system detect the location of the flame’s focus thanks to the models of the algorithm explained in the previous section. Nevertheless, with the increasing advance in neural networks applied over image processing, it is possible to train a deep neural network that is capable of detecting fire [15] in thermal images with our developed method, which improves the processing time and reduces the false positives below 1% and the false negatives below 2%. Although most solutions usually use RGB cameras, we successfully validated our indoor spaces algorithms with no visibility. Our algorithm allows us to detect the beginning of the emergency’s focus when the level of the fire is imperceptible using images with a high level of smoke. Moreover, most of the works make their proposed static and security cameras, making detection easier. However, in this work, we have deployed and evaluated the system using a moving camera.

For this reason, our system has detection metrics lower than 90% because the moving camera complicates the results. Additionally, the focus of this type of camera is static, which has made deployment difficult. Moreover, we have deployed and evaluated the processing to answer in real time in an embedded system with computational limitations, optimizing the results to operate in the proposed scenarios.

Related to sensor networks, the sensors previously deployed have certain advantages, such as knowledge of exact locations and the ability to provide data from regions that may be occluded due to closed doorways or structural collapse. On the other hand, the point-of-emergency deployment capability and mobility of sensor nodes allow larger deployments and higher resolution sensing of spaces to be introduced [11,43]. In those spaces, the location of a point of interest related to the emergence could improve the results. Bluetooth Low Energy (BLE) is mentioned in the next surveys [12], sufficient to relay low-bandwidth data. Nevertheless, more complex communication scenarios with heterogeneous sensor nodes with varying bandwidth and distance requirements are revised; indeed, the network must provide quality-of-service for both higher-bandwidth data such as camera streams as well as low-bandwidth temperature readings. Another challenge is the co-existence of delay-tolerant communication with communication that has time requirements, such as what is required for node localization. Furthermore, nodes’ mobility can provide opportunities in designing routing and discovery schemes better suited to such networks.

Our proposal can operate optimally and efficiently under dense smoke, and flames are hazardous. Most papers on state-of-the-art techniques reflect that these are validated in simulated environments, and there are no solutions with terminal images and a sensor network to detect and locate the focus at an emergency in indoor spaces. We validated the proposal in real environments with no GNSS signals, in indoor spaces, and no visibility. The optimal route could be for a single-store building or entire complex buildings, or large basements, which will enhance the emergency. Our algorithm allows us to detect the beginning of the emergency’s focus when the level of fire is imperceptible. This result allows for a faster response for security personnel. We conclude that this kind of location-aware fire integrated systems will have a meaningful impact on first responder interventions’ speed and security.

## 7. Conclusions

In this paper, we described a system to solve the scientific-technological challenges and improve the available tools for Emergency Response Teams in indoor and hostile environments without visibility and lack of GNSS signal and communications in fire emergencies (initial stage or advanced). The proposal consists of a sensor network (beacons), a Mobile Ground Autonomous Vehicle (MGAV), and a mobile application (monitoring interface) that has been implemented, deployed, and evaluated. The platform allows offering communications and environmental information from inside to outside. The detection system algorithm can detect the fire points from thermal images with great accuracy (0.73% accuracy and 0.99% precision) in two real scenarios with hot and cold smoke. We will study other intents with fire and the possibility of having a repository of different adjustment parameters for different environments or some self-adjustment strategy. This computer vision algorithm for fire detection has the capability of operating in low visibility conditions. The fire detection algorithm performs a temporal analysis and color filtering to detect candidate fire regions in the image. Afterward, it extracts characteristics that facilitate and allow the discrimination of fire from other similar regions. Moreover, a linear interpolation was used in order to reduce the number of pixels for being processed. We applied Gaussian smoothing, which allows the images to be transformed into the HSV color space to reduce noise.

Moreover, the contrast of the image has been improved by combining two techniques CLAHE and ImageEnhace. In the second stage, the candidate regions’ segmentation is performed considering that the fire will move from one image to another. In the last stage, the algorithm stores the regions’ characteristics classified as fire with their outlines and sends them to the monitoring interface.

The combined use of the fire detection system and the environmental sensor network, the first-responder localization, and a set of edge computing algorithms allow improving the firefighter tactics during an emergency intervention and the evacuation from a hostile site. These provide valuable information in an emergency intervention and its impact on the firefighter response times and operational timeframes.

For future work related to this paper, considering a more extensive communications range than a star network, a Mesh Network for the robustness of the communications could be proposed. Therefore, the next step would be to evolve to a mesh network topology and have a complete connection between all the nodes. The type of sensorization proposed in the paper is a trend towards intelligent buildings in the future, with IoT sensors (temperature, light, gas concentrations, fire, etc.). For high-rise buildings, the solution has to be adapted. However, in emergency scenarios, having preventive information can help to improve the intervention. The more precise the relevant points of interest in the emergency are, the better and more effective the results will be. In this work, we focused on a single space without physical references. We are improving our research for a structured scenario with walls to discriminate the different rooms’ states. Therefore, it is expected that we can deploy this type of network at much lower costs, as the economy of scale between production chains and regulations requires building sensorization. Furthermore, to improve the interface, a possible future work can be to guide the intervening team personnel using ultrasounds or visual signals.

## Figures and Tables

**Table 1:** Real RRSI results obtained in the test.

Static Parameters		Tx = 8 dB				Tx = 0 dB				Tx = −8 dB			
		RSSI (dBm)				RSSI (dBm)				RSSI (dBm)			
		B1	B2	B3	B4	B1	B2	B3	B4	B1	B2	B3	B4
Average	Smokeless	−58.83	−59.82	−63.00	−58.18	−66.36	−67.18	−71.45	−63.00	−73.27	−76.09	−75.27	−74.09
	With smoke	−57.55	−56.13	−58.37	−56,21	−65.27	−65.27	−68.95	−65.64	−72.64	−70.84	−74.91	−73.59
Mode	Smokeless	−62	−55	−64	−60	−69	−71	−	−69	−75	−80	−79	−77
	With smoke	−66	−	−56	−59	−70	−71	−71	−68	−	−81	−73	−
Median	Smokeless	−62	−59	−64	−60	−69	−69	−73	−65	−75	−80	−79	−77
	With smoke	−61	−57	−57	−58	−67	−65	−71	−68	−75	−72	−75	−74
Maximum	Smokeless	−70	−72	−74	−64	−78	−79	−80	−70	−82	−85	−88	−86
	With smoke	−66	−66	−71	−62	−76	−82	−82	−73	−84	−82	−89	−84
Standard deviation	Smokeless	9.90	7.17	8.51	6.1	10.77	8.84	7.09	6.12	8.49	8.97	13.47	8.10
	With smoke	11.20	8.74	5.63	5.81	10.72	10.09	8.01	8.43	9.11	10.35	9.99	10.38
Variance	Smokeless	98.04	51.36	72.40	37.16	116.05	78.16	50.27	37.40	72.02	80.49	181.,42	65.69
	With smoke	125.47	76.35	31.67	33.76	114.82	101.82	64.12	71.05	83.05	107.03	99.89	107.84

**Table 2:** Real Location of the beacons and points.

**Beacons**	**Position on the x-Axis (m)**	**Position on the y-Axis (m)**	**Points**	**Position on the x-Axis (m)**	**Position on the y-Axis (m)**
			P1	1.63	4.61
			P2	1.63	10.35
B1	0	3.3	P3	5.74	10.35
B2	0	9.6	P4	5.74	5.2
B3	5.06	11.89	P5	3.35	5.2
B4	6.38	7	P6	3.35	8.12

**Table 3:** Characteristics of the video made in the CUS and the fire detection results obtained by applying the video’s algorithm.

Video Characteristics	Duration (Minutes)	Frames (Total Number)	Resolution (Pixels)	Image Type
	1:40	1.000	640 × 512	Original
Detection metrics	Accuracy (%)	Precision (%)	Recall (%)	F1 Score (%)
	79%	94%	74%	83%
Processing time	No optional parameters		With optional parameters	
	Complete video (s)	1 frame (s)	Complete video (s)	1 frame (s)
	43	0.69	73	0.80

**Table 4:** RSSI statistical parameters per beacon.

Static Parameters		RSSI (dBm)			
		Beacon 1	Beacon 2	Beacon 3	Beacon 4
Average	With smoke	−70.20	−75.92	−78.16	−79.96
	Smokeless	−72.73	−76.54	−80.32	−82.00
Mode	With smoke	−70	−75	−76	−77
	Smokeless	−76	−73	−82	−82
Median	With smoke	−70	−75	−78	−80
	Smokeless	−73	−76	−81	−82
Maximum	With smoke	−82	−84	−85	−84
	Smokeless	−79	−84	−84	−85
Standard deviation	With smoke	5.11	4.26	3.2	2.34
	Smokeless	4.07	3.89	2.7	2.04
Variance	With smoke	26.08	18.16	10.56	5.46
	Smokeless	16.60	15.13	6.14	4.17

**Table 5:** Vision algorithm results for fire detection.

Complete Video in Teresa				
Video	Duration	Total Number of Frames	Processing Time	False Positives
Complete Video	8 min: 20 s	15.000 frames	42 min	102 frames 0.68%
**Clippings of the Video in Teresa.**				
**Video**	**Duration**	**Total Number of Frames**	**Processing Time**	
			**Without Fire**	**With Fire**
Clip 1	10 s	300 frames	1 min: 18 s	54 s
Clip 2	30 s	900 frames	3 min: 59 s	2 min: 49 s
Clip 3	45 s	1.350 frames	6 min: 11 s	4 min: 45 s
Clip 4	60 s	1.800 frames	6 min: 45 s	6 min: 26 s

**Table 6:** Characteristics of the video made in Teresa and the fire detection results obtained by applying the algorithm on the video.

**Video Characteristics**	**Duration (Min)**	**Frames (Total Number)**	**Resolution (Pixels)**	**Image Type**
	1:00	1.800	640 × 512	Rainbow
**Detection metrics**	**Accuracy (%)**	**Precision (%)**	**Recall (%)**	**F1 Score (%)**
	0.73	0.99	0.70	0.82
**Processing time**	**No optional Parameters**		**With Optional Parameters**	
	**Complete Video (s)**	**1 Frame (s)**	**Complete Video (s)**	**1 Frame (s)**
	76	0.66	128	0.83

**Table 7:** The position of the point in the map followed the Beacons to the fire focus.

Point	Firefighter Position on the x-Axis (m)	Position on the y-Axis (m)	Error (m)	Point	Position on the x-Axis (m)	Position on the y-Axis (m)	Error (m)
0	0	0	0	9	1.195	5.652	0.165
1	0.548	0.582	0.012	10	1.420	6.420	0.050
2	1.142	1.118	0.007	11	1.407	7.220	0.050
3	1.570	1.794	0.273	12	1.369	8.019	0.100
4	1.097	2.439	0.218	13	1.336	8.818	0.141
5	0.701	3.134	0.256	14	1.302	9.617	0.133
6	0.023	3.558	0.094	15	0.697	10.141	0.036
7	0.423	4.251	0.330	16	0.093	10.666	0.195
8	0.803	4.955	0.229	17	−0.521	11.178	0.129

**Table 8:** Comparison of main contributions.

	Environmental Parameters	Image Processing for Flame/Fire Focus Detection Thermal Vision	Location Estimation for Navigation and Firefighter Tracking			
			Navigation and Tracking	Indoor and Simulated	Indoor and Real Scenario Evaluation	Modular
Our proposal	Yes	Yes	Yes	Yes	Yes	Yes
FREAS [11]	No	Yes	No	No	Yes	No
“FireBack” [9]	Yes	No/Yes	Only See in the map	No	No	No
“A survey” [12]	No	No	Yes	Yes	Yes	
CROW [14]	No	No	Yes	No	Yes	No
ICA K-medoids [16]	No	Yes, but only with RGB images	No	Only outdoor without smoke	Only outdoor without smoke	No
“Accurate” [26]	No	No	Yes	No	Yes	No
“Potentialities” [45]	No	Yes/No	No	No	Yes	No
SensorFly [46]	Yes	No/Yes	No	Yes	Yes	No

## Data Availability

Not applicable.
